# Role of the Skin Microenvironment in Melanomagenesis: Epidermal Keratinocytes and Dermal Fibroblasts Promote BRAF Oncogene-Induced Senescence Escape in Melanocytes

**DOI:** 10.3390/cancers14051233

**Published:** 2022-02-27

**Authors:** Shreyans Sadangi, Katarina Milosavljevic, Edgardo Castro-Perez, Marcos Lares, Mithalesh Singh, Sarah Altameemi, David J. Beebe, Jose M. Ayuso, Vijayasaradhi Setaluri

**Affiliations:** 1 Department of Dermatology, University of Wisconsin-Madison, Madison, WI 53705, USA; sadangi@wisc.edu (S.S.); milosavljevi@wisc.edu (K.M.); ecastro@indicasat.org.pa (E.C.-P.); mlares@dermatology.wisc.edu (M.L.); msingh@dermatology.wisc.edu (M.S.); saltameemi@dermatology.wisc.edu (S.A.); 2 Department of Pathology and Laboratory Medicine, University of Wisconsin, 1111 Highland Ave., Madison, WI 53705, USA; djbeebe@wisc.edu; 3 Department of Biomedical Engineering, University of Wisconsin-Madison, 1550 Engineering Drive, Madison, WI 53706, USA; 4 The University of Wisconsin Carbone Cancer Center, University of Wisconsin-Madison, Madison, WI 53706, USA

**Keywords:** melanoma, tumor microenvironment, senescence, microfluidics

## Abstract

**Simple Summary:**

Melanoma is a deadly skin cancer caused by the uncontrolled proliferation of melanocytes, a population of specialized cells that produce the skin pigment melanin. An aberrant proliferation of melanocytes is common, manifesting as new moles, and these lesions often remain benign. Only a small fraction of these aberrant melanocytes transition to melanoma (i.e., melanomagenesis). The factors that drive this transition are not fully understood. Recent studies have suggested that other cells—specifically, keratinocytes that make up the upper skin layers and fibroblasts, which are non-specialized cells within the deeper layers of the skin—also contribute to melanomagenesis. Here, employing microscale models that mimicked the skin microenvironment, we investigated the effect of crosstalk between melanocytes as well as keratinocytes and fibroblasts on melanomagenesis. Our findings show that keratinocyte- and fibroblast-derived factors can inhibit the mechanisms that prevent an uncontrolled melanocyte proliferation and contribute to melanomagenesis. Thus, targeting skin microenvironment-derived factors is a potential strategy to prevent melanomagenesis.

**Abstract:**

*BRAF^V600E^* is the most common mutation driver in melanoma. This mutation is known to cause a brief burst of proliferation followed by growth arrest and senescence, which prevent an uncontrolled cell proliferation. This phenomenon is known as oncogene-induced senescence (OIS) and OIS escape is thought to lead to melanomagenesis. Much attention has been focused on the melanocyte-intrinsic mechanisms that contribute to senescence escape. Additional genetic events such as the loss of tumor suppressor *PTEN* and/or epigenetic changes that contribute to senescence escape have been described. However, the role of the skin microenvironment—specifically, the role of epidermal keratinocytes—on melanomagenesis is not fully understood. In this study, we employ a microfluidic platform to study the interaction between melanocytes expressing the *BRAF^V600E^* mutation as well as keratinocytes and dermal fibroblasts. We demonstrate that keratinocytes suppress senescence-related genes and promote the proliferation of transformed melanocytes. We also show that a keratinocyte-conditioned medium can alter the secretion of both pro- and anti-tumorigenic factors by transformed melanocytes. In addition, we show that melanocytes and keratinocytes from donors of white European and black African ancestry display different crosstalks; i.e., white keratinocytes appear to promote a more pro-tumorigenic phenotype compared with black keratinocytes. These data suggest that keratinocytes exert their influence on melanomagenesis both by suppressing senescence-related genes in melanocytes and by affecting the balance of the melanocyte-secreted factors that favor tumorigenesis.

## 1. Introduction

Melanoma is one of the most common malignancies in the U.S. [1] and melanoma incidence has more than tripled in the past three decades (1975–2020). The American Cancer Society estimates that more than 100,000 new melanoma cases will be diagnosed in 2021 with nearly 8000 related deaths in the U.S. [1]. Cutaneous melanoma arises from melanocytes, the pigment-producing cells located in the skin [2]. The most common mutation drivers found in melanoma are the activating mutations in the *BRAF* gene. About 60% of melanoma tumors harbor the *BRAF^V600E^* mutation, which leads to a constitutive activation of the mitogen-activated protein kinase (*MAPK*) pathway [3,4]. After an initial burst of proliferation, melanocytes that acquire the oncogenic driver mutation enter a state of growth arrest known as oncogene-induced senescence (OIS) [5]. The observation that the majority of nevi, which are a collection of senescent melanocytes, also harbor the *BRAF^V600E^* mutation supports this notion of OIS [4]. OIS is thought to act as a mechanism by which transformed melanocytes are prevented from a runaway proliferation leading to tumorigenesis. 

Accordingly, OIS escape is considered to be a prerequisite step in melanomagenesis. In this context, several mechanisms that promote OIS escape/bypass have been identified. For instance, a loss of the tumor suppressor gene *PTEN* results in the suppression of senescence in melanocytes carrying the *BRAF^V600E^* mutation [6]. The deletion of *PTEN* in mutant BRAF nevi has also been reported to drive tumor development in vivo.

Epigenetic events have also been implicated in the abrogation of OIS. H3K9 demethylases *LSD1* and *JMJD2C* were shown to not only promote a *BRAF* oncogene-induced senescence bypass but also reverse senescence and promote cell proliferation [7]. Although most studies have focused on the melanocyte-intrinsic molecular events that contribute to OIS escape, the role of the skin microenvironment—specifically, epidermal keratinocytes and dermal fibroblasts—on OIS escape has not been adequately investigated. These cells interact with melanocytes via the paracrine [8,9,10] and cell–cell interaction [11,12], thus potentially regulating melanoma initiation and progression. An active crosstalk between keratinocytes and melanocytes and its role in skin pigmentation is well-documented. Keratinocytes secrete factors such as melanocyte-stimulating hormones that facilitate the synthesis of melanin in melanocytes [13,14]; melanin-containing melanosomes are then transported back to keratinocytes where they protect DNA from UV damage [15,16]. This interaction is more pronounced in response to UV exposure. We have also seen the ability of keratinocytes to modify melanoma cell morphology in vitro [17] and decrease Tyr expression, a key player in pigmentation and the control of the Ink4A-Arf locus of genes [18]. Based on these observations, we hypothesized that epidermal keratinocytes impact OIS in melanocytes. To test this, we employed microfluidics, which offers a robust platform to co-culture multiple cell types in the desired pattern and monitor cellular [19,20] and microenvironmental crosstalk [21,22,23]. Due to the significantly lower volume of media required in microfluidic devices (typically in the µL scale) compared with traditional Petri dishes and multi-well plates, these systems are well-suited to mimic the cell response in vivo in the context of the tissue architecture and concentrations of the secreted factors [24,25].

In this study, we set out to investigate the effect of keratinocytes on the senescence induced by the *BRAF^V600E^* mutation. We monitored the senescence-associated β-galactosidase (SA-β-gal) activity, the expression of senescence-related genes in melanocytes, melanocyte secretome and the effect of keratinocyte secretome to evaluate OIS in mutant *BRAF*-transduced melanocytes in the presence of skin stromal cells. We show that keratinocytes altered the transcriptomic profile of melanocytes, particularly impacting on the genes that are key players in senescence and cell proliferation.

## 2. Results

### 2.1. BRAF^V600E^ Transformation Induces a Growth Arrest and Senescence after an Initial Burst of Proliferation in Normal Human Melanocytes

**Figure 1:**
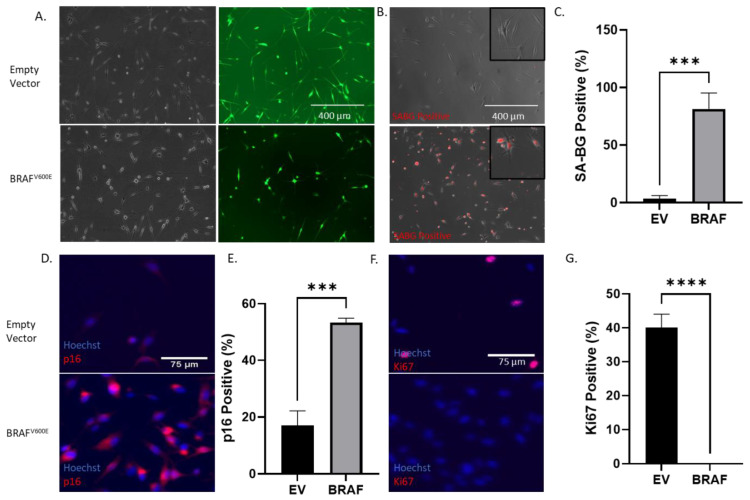
*BRAF^V600E^* induces growth arrest in melanocytes. (**A**) Upper panel shows Caucasian melanocytes transduced with the control lentivirus carrying an empty vector coding for green fluorescence protein (GFP). Lower Panel shows Caucasian melanocytes transduced with the *BRAF^V600E^* mutant lentivirus (60% transduction efficiency). (**B**) Senescence-associated beta-galactosidase (SA-β-gal) staining of Caucasian melanocytes after transduction with a *BRAF^V600E^* lentivirus. Upper panel shows transduction with the empty vector whereas the lower image shows *BRAF^V600E^*-transduced melanocytes after the addition of the beta-gal substrate, DCM beta-gal. Insets show magnified images. (**C**) Bar graph shows the percentage of SA-β-galactosidase (SA-β-gal)-positive cells shown in (**B**). (**D**,**E**) Immunofluorescence (IF) images showing p16 staining, a cell cycle inhibitor commonly upregulated during senescence. *BRAF^V600E^*-transduced melanocytes (lower image) show a higher expression of p16 than control melanocytes (upper image). (**F**,**G**) IF images showing the cell proliferation marker Ki67. *BRAF^V600E^*-transduced melanocytes (lower image) show no expression of Ki67, suggesting the growth arrest of cells. Experiments were repeated at least 3 independent times. Data were analyzed using a Student’s *t*-test with a Welch correction. Graphs show mean ± standard deviation. *** denotes *p* < 0.005; **** denotes *p* < 0.0005.

We transduced primary human melanocytes with the mutant *BRAF^V600E^* lentivirus (Figure 1A) and evaluated the expression of the markers for senescence (SA-β-gal and p16) and cell proliferation (Ki67).

The data in Figure 1B,C show that melanocytes expressing *BRAF^V600E^* showed an elevated SA-β-gal activity and p16 expression (Figure 1D,E) and a decreased expression of Ki67 (Figure 1F,G). We also noted the induction of p27, another senescence marker, in the oncogenic melanocytes (Figure S1A). We also observed the formation of senescence-associated heterochromatin foci (SAHF), a distinct chromatin organization found in senescent cells [26], as evidenced by the punctate staining of the nuclei in the *BRAF^V600E^*-transformed melanocytes (Figure S1B). These results demonstrated that the *BRAF^V600E^* oncogene induced OIS and growth arrest in human primary melanocytes. Due to the difference in the cell proliferation rate between the mutant *BRAF*-transduced and empty vector-transduced melanocytes, we seeded melanocytes in all subsequent experiments at a cell density that ensured none of the culture conditions analyzed reached a confluency before finishing the experiment.

### 2.2. Keratinocytes Suppress Oncogene-Induced Senescence in BRAF^V600E^ Melanocytes

**Figure 2:**
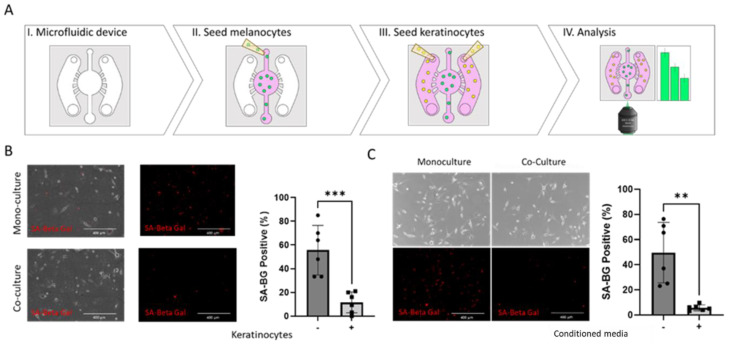
Effect of keratinocytes on melanocyte oncogene-induced senescence (OIS). (**A**) Schematic of the melanocyte and keratinocyte co-culture. Melanocytes were seeded in the central chamber and keratinocytes in the lateral chamber. The microdevice design allowed us to confine each cell type into the desired chamber whilst allowing cell–cell crosstalk. Cells were analyzed for SA-β-gal activity 7 days after co-culture. (**B**) *BRAF^V600E^*-transduced Caucasian melanocytes were seeded on the central chamber of the microfluidic device and cultured with autologous keratinocytes (+) or culture media only (-) in the lateral chambers. Images show the SA-β-gal red signal overlaid with a brightfield. We observed that the presence of keratinocytes lowered the number of oncogene-induced senescence (OIS)-positive *BRAF^V600E^* melanocytes. (**C**) *BRAF^V600E^*-transduced Caucasian melanocytes were seeded on a 96-well plate and cultured with autologous Caucasian keratinocyte-conditioned media or fresh keratinocyte media. Left and right panels show melanocytes in phase contrast in media and keratinocyte-conditioned media, respectively, suggesting a keratinocyte-induced OIS decrease. Melanocytes were 12 days post-transduction and 7 days after co-culture. ** denotes *p* < 0.005; *** denotes *p* < 0.0005. Experiments were repeated at least 3 independent times. Data were analyzed using a Student’s *t*-test with a Welch correction. Graphs show mean ± standard deviation.

In the context of OIS escape, the role of the skin microenvironment—specifically, the influence of keratinocytes—is not fully understood. We co-cultured mutant *BRAF* lentivirus-transduced melanocytes with human primary keratinocytes in a microfluidic device to study the crosstalk between keratinocytes and melanocytes in the context of OIS (Figure 2A, I–IV). We seeded the transformed melanocytes in the central chamber of the microdevice and the autologous keratinocytes on the flanking chambers. The cells remained confined in the respective chambers during the seeding process but were allowed to communicate via the connecting channels.

We cultured the cells for seven days and monitored the SA-β-gal activity. As shown in Figure 1, a mutant *BRAF* expression induced OIS and growth arrest. However, the presence of keratinocytes in the adjoining chambers resulted in fewer senescent cells (Figure 2B).

As this microfluidic device allowed a two-way crosstalk, we exposed *BRAF^V600E^* melanocytes to a conditioned medium from autologous keratinocytes to investigate the role of keratinocyte-secreted factors alone on the transformed melanocytes. Similar to the results in the device that allowed a two-way communication, we observed that keratinocyte-conditioned media suppressed senescence in the transformed melanocytes (Figure 2C). 

This validated the role of keratinocyte-secreted factors in OIS bypass in melanocytes. We also studied the effect of a keratinocyte co-culture in a transwell assay and showed that keratinocytes suppressed senescence in *BRAF^V600E^*-transduced melanocytes (Figure S2A). Additionally, we co-cultured melanocytes and keratinocytes in a transwell plate where we seeded mutant *BRAF*-transduced melanocytes in the bottom well of the transwell plate and seeded autologous keratinocytes in the insert. The results showed a similar reduction in the p16 expression of the transformed melanocytes in the presence of keratinocytes (Figure S2B), suggesting that paracrine signaling contributed to the keratinocyte-induced OIS escape. We then examined the role of fibroblasts in OIS escape by co-culturing *BRAF*-transduced melanocytes and dermal fibroblasts in the microfluidic device. We co-cultured melanocytes with autologous dermal fibroblasts and observed a similar effect of fibroblasts on OIS reduction and melanocyte morphology as seen with keratinocytes (Figure S2C). Taken together, these results demonstrated that the stromal cells present in the skin microenvironment could suppress OIS in transformed melanocytes and promote a malignant phenotype via paracrine signaling.

### 2.3. Keratinocytes Condition OIS-Associated Gene Expression in Primary Melanocytes

**Figure 3:**
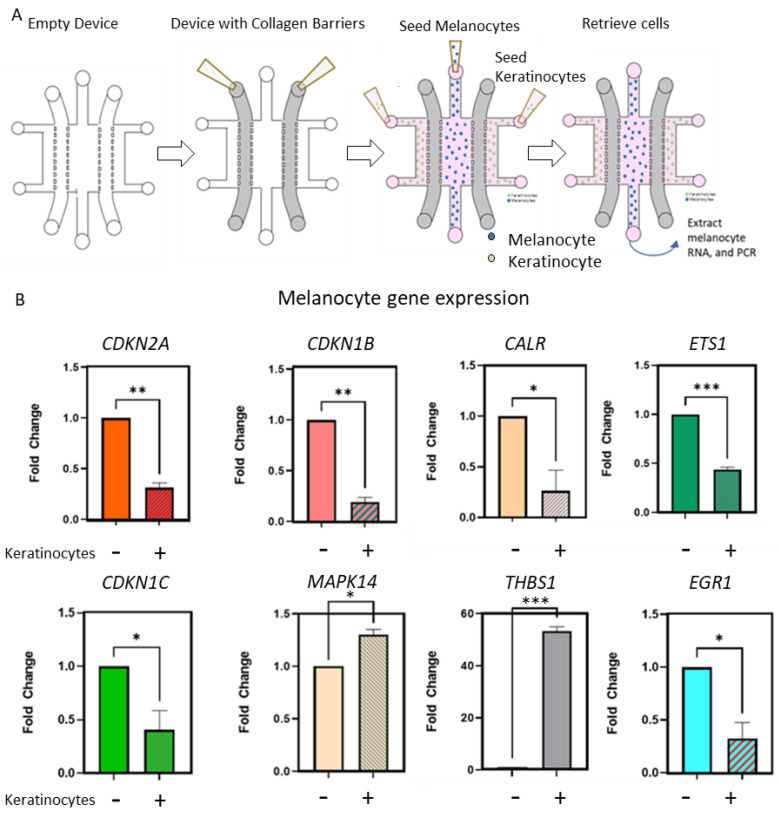
Transcriptomic changes due to the presence of Caucasian ancestry keratinocytes. (**A**) Schematic showing the co-culture of Caucasian ancestry melanocytes and keratinocytes. A collagen mixture was added to the second and fourth chambers and allowed to polymerize (approximately 15 min). Transformed melanocytes (5 days post-transduction) were seeded in the central chamber and keratinocytes were seeded in the extreme lateral chambers. Cells were co-cultured for 7 days following which RNA was extracted and followed with a PCR analysis. (**B**) Bar graphs showing a fold change of selected genes in the absence (−) and presence (+) of keratinocytes. * denotes < 0.05; ** denotes < 0.005; *** denotes < 0.0005. Data were analyzed using a Student’s *t*-test with a Welch correction. Graphs show mean ± standard deviation.

We then investigated the gene expression changes that contributed to a keratinocyte-induced OIS escape. In order to specifically isolate melanocytes after the co-culture with keratinocytes, we employed another microdevice that allowed the selective retrieval of melanocytes without the contamination of keratinocytes from the adjacent chambers (Figure 3A). For this, we modified our microdevice to include a collagen hydrogel barrier to confine the cells in their respective chambers whilst allowing cell–cell communication by the secreted factors. After a seven day co-culture with keratinocytes, we retrieved melanocytes by the selective trypsinization of the central microchamber and analyzed the senescence-related genes by a RT-qPCR analysis. Our analysis revealed that the presence of keratinocytes resulted in the downregulation of the cell cycle regulatory genes *CDKN1B, CDKN1C, CDKN2A* and *CDKN2C* that code for p27, p57, p16 and p18, respectively. These proteins function as cell cycle inhibitors and also as tumor suppressors (Figure 3B) [27,28,29,30,31]. Keratinocytes also upregulated *MAPK14*, a component of the MAP kinase pathway associated with an increased cell proliferation. In addition to the genes directly related to the cell cycle and proliferation, we noted the downregulation of several genes such as *CALR1, ETS1* and *EGR1* that are associated with senescence (Figure 3B), for example, Calreticulin (*CALR1*), a Ca^2+^ binding protein that is upregulated in senescent cells and reported to be involved in the recruitment of macrophages for the phagocytosis of senescent cells [32]. *ETS1* is a transcription factor that has been shown to drive senescence by regulating the p16 expression in human fibroblasts [33,34]. Similarly, *EGR1* is known to act as a tumor suppressor by regulating growth and differentiation, as evidenced by a complete senescence bypass and the immortalization of *EGR1*-null mouse embryonic fibroblasts [35]. We also noted approximately a 50-fold increase in the expression of thrombospondin (*THSB1*) in melanocytes in the presence of keratinocytes.

*THSB1* has been shown to regulate OIS escape and also contribute to a more invasive phenotype of melanoma [36,37]. We also observed changes in other genes associated with additional cell growth regulatory functions (e.g., *TGF-β*) (Figure S3), highlighting the complex response elicited by the presence of keratinocytes. Overall, these data demonstrate that keratinocytes altered the expression of multiple genes associated with senescence and promoted the OIS escape of mutant *BRAF*-transformed melanocytes. In this study, we limited the gene expression analysis to senescence-related genes. However, future studies could leverage the power of whole-transcriptome and single-cell RNA sequencing that will allow a complete understanding of the molecular changes induced in melanocytes by the skin microenvironment. 

### 2.4. BRAF^V600E^ Transformation and Keratinocytes Alter Growth Factor Secretion in Melanocytes

**Figure 4:**
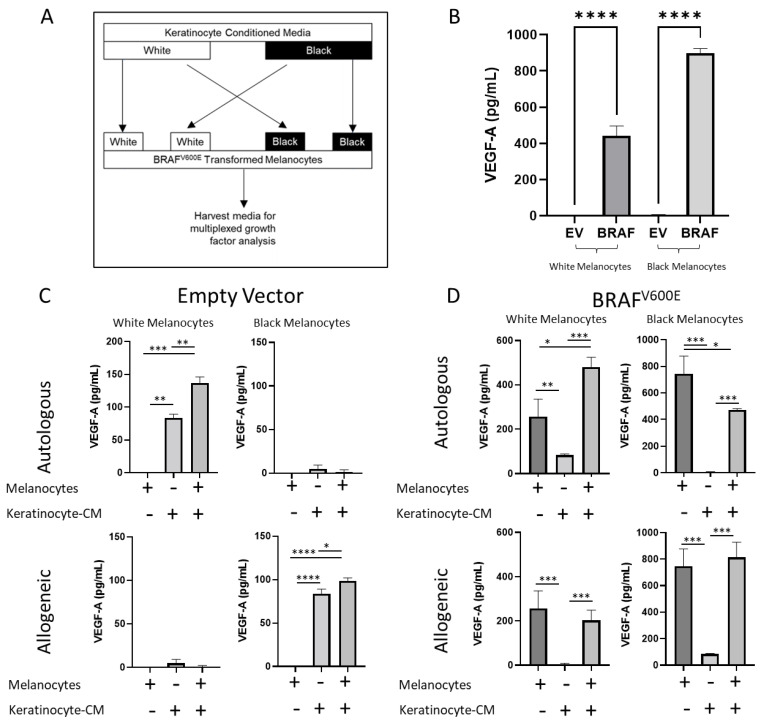
VEGF-A secretion is modulated by *BRAF^V600E^* expression and keratinocytes. (**A**) Experimental design. Transformed and untransformed Caucasian melanocytes were exposed to a keratinocyte-conditioned medium from autologous and allogenic keratinocytes for 3 days and the spent media were harvested for a growth factor analysis. (**B**) Effect of oncogene transformation on VEGF-A secretion. *BRAF^V600E^* expression enables the secretion of VEGF-A by melanocytes in isolation. (**C**) Effect of keratinocytes on VEGF-A secretion by normal melanocytes. Caucasian keratinocytes enable the secretion of VEGF-A in normal melanocytes in autologous and allogeneic melanocytes. (**D**) Effect of keratinocytes on VEGF-A secretion by transformed melanocytes. African-American keratinocytes suppress VEGF-A secretion by transformed African-American melanocytes. + in (**C**) and (**D**). * denotes *p* < 0.05; ** denotes *p* < 0.005; *** denotes *p* < 0.0005; **** denotes *p* < 0.00005. Experiments were repeated at least 3 independent times. Data were analyzed using a Student’s *t*-test with a Welch correction. Graphs show mean ± standard deviation.

We then asked whether keratinocytes also affect the secretome of oncogenic melanocytes in addition to the gene expression changes in melanocytes. Several secreted factors in the melanocyte microenvironment are also known to promote tumor development, cell invasion and metastasis. We also investigated whether differences in the action of white Caucasian (European ancestry) and black African-American (African ancestry) keratinocytes on the transformed melanocytes could explain, in part, the disparity of melanoma incidence in light- and dark-skinned individuals [38]. We performed a targeted proteomic analysis to identify the growth factors secreted by melanocytes in the presence of keratinocyte-conditioned media (Figure 4A). Among the 11 factors analyzed, we observed an increased *VEGF-A* secretion, which has been implicated in tumor-induced angiogenesis, melanoma progression [39] and resistance to *BRAF* inhibitors [40]. We observed that both Caucasian and African-American keratinocytes secreted *VEGF-A*, with Caucasian keratinocytes secreting higher levels (Figure S4). Normal melanocytes by themselves did not secrete *VEGF-A* but when co-cultured with keratinocyte-conditioned media, there was an increase in *VEGF-A* in the medium. Mutant *BRAF*-transduced melanocytes by themselves appeared to secrete a higher amount of *VEGF-A* compared with normal melanocytes isolated from both light- and dark-skinned individuals (Figure 4B,C). Interestingly, autologous and non-autologous keratinocytes did not affect the secretion of *VEGF-A* by oncogene-transformed Caucasian melanocytes. These data suggest that although keratinocytes can stimulate the secretion of factors such as *VEGF-A* by their normal counterparts, malignant melanocytes appear to become refractory to this stimulation (Figure 4D).

We observed a decrease in VEGF-A secretion in the African-American-transformed melanocytes when exposed to an autologous keratinocyte-conditioned medium. We also investigated the effects of allogeneic keratinocytes on normal and transformed melanocytes. We found that Caucasian keratinocytes stimulated VEGF-A secretion in African-American melanocytes as they did with their autologous counterparts, suggesting that keratinocytes from Caucasian skin may contribute to tumor growth and are spread by creating a more pro-angiogenic environment.

**Figure 5:**
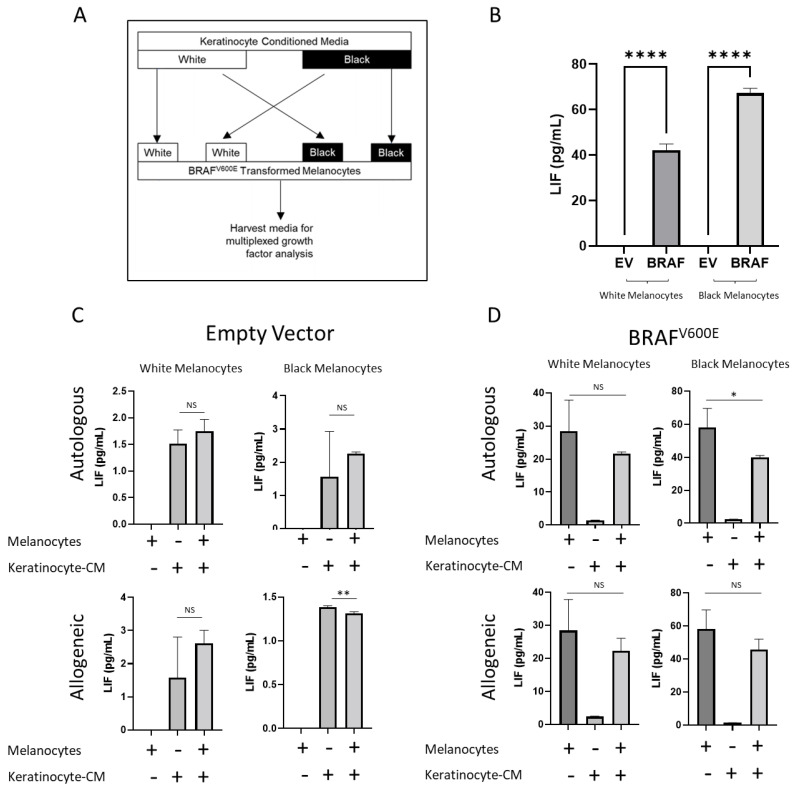
LIF secretion is modulated by *BRAF^V600E^* expression and keratinocytes. (**A**) Experimental design. Transformed Caucasian melanocytes were exposed to autologous and allogeneic keratinocytes for 3 days and the spent keratinocyte-conditioned medium was harvested for a growth factor analysis. (**B**) Effect of oncogene transformation on LIF. *BRAF^V600E^* expression enables the secretion of VEGF-A by melanocytes in isolation. (**C**) Effect of keratinocytes on LIF secretion by normal melanocytes. As opposed to VEGF-A, keratinocytes had a minor effect on LIF secretion by normal melanocytes. (**D**) Effect of keratinocytes on LIF secretion by *BRAF*-transformed melanocytes. Similar to the empty vector, keratinocytes showed a minor effect on LIF secretion by *BRAF*-transduced melanocytes. * denotes *p* < 0.05; ** denotes *p* < 0.005; **** denotes *p* < 0.00005. Experiments were repeated at least 3 independent times. Data were analyzed using a Student’s *t*-test with a Welch correction. Graphs show mean ± standard deviation. NS denotes non-significant difference.

The leukemia inhibitory factor (LIF) secreted by melanocytes was also regulated by the presence of African-American keratinocytes (Figure 5A). LIF has been shown to be strongly expressed in melanoma cell lines and contributes to increased attachment, proliferation and migration. The leukemia inhibitory factor receptor (LIFR) has been associated with a worse outcome in melanoma patients [41,42].

We observed a similar pattern in the LIF secretion as observed with VEGF-A. Normal melanocytes did not secrete LIF but upon an oncogenic transformation, they began to secrete LIF (Figure 5B). We noted that African-American keratinocytes decreased the LIF secretion in allogeneic Caucasian melanocytes (Figure 5C,D) whereas Caucasian keratinocytes did not affect LIF secretion. Overall, these results showed that keratinocytes exert a complex effect on *BRAF^V600E^*-transformed melanocytes, exhibiting differences between Caucasian and African-American keratinocytes. In this study, we demonstrated that African-American and Caucasian keratinocytes condition melanocytes in a different fashion. However, the overall effect of this differential crosstalk requires in-depth studies that leverage RNA-seq or proteomics to perform large screenings to identify all the molecular pathways involved. 

Taken together, our data showed that the dynamic interaction between keratinocytes and normal melanocytes in healthy skin operate to promote melanomagenesis by both melanocyte- and keratinocyte-secreted factors as well as keratinocyte-induced gene expression changes in nascent transformed melanocytes.

## 3. Discussion

The transition from melanocytes to melanoma formation is a complex process. Previous studies have demonstrated that the skin microenvironment modulates melanomagenesis through numerous and complex processes involving environmental and cellular signals [1,2]. OIS is widely considered to be a protective mechanism; escape from this putative growth arrest is thought to be one of multiple events that drive tumorigenesis. Thus, determining what causes oncogenic melanocytes to escape this innate defense is important to understand the early events of melanomagenesis. Studies have shown that additional genetic or epigenetic changes can facilitate or suppress OIS [6,7]. The effect of the skin microenvironment on OIS in melanocytes has not been completely investigated. The skin microenvironment, which mostly harbors keratinocytes and dermal fibroblasts, has been shown to modify the behavior of both oncogenic melanocytes and the primary tumor behavior [17]. Therefore, in this study we asked if the skin niche could also influence OIS and attempted to determine the possible molecular and genetic changes that contribute to this effect. We used a microfluidic platform to model the skin microenvironment and showed that keratinocytes and fibroblasts suppressed OIS in melanocytes via paracrine factors. We also demonstrated genetic changes in the transformed melanocytes in the presence of keratinocytes, which showed altered expression of the genes that regulate cell proliferation and characterize the senescence phenotype. In this study, we limited our gene expression analysis to a few selected genes. However, future studies could leverage the power of RNA sequencing and other genomic techniques in combination with microfluidic devices to perform a non-supervised whole exome analysis. This approach would allow researchers to decipher the molecular changes induced in melanocytes by the presence of skin stromal cell-like keratinocytes and fibroblasts, which in turn could offer new therapeutic windows targeting these pathways. 

Collectively, the results showed that keratinocytes could suppress oncogene-induced senescence (OIS) in the transformed melanocytes. As OIS is thought to be a fail-safe mechanism to prevent the runaway proliferation of transformed cells, our results suggested that keratinocytes might inhibit or help melanocytes overcome this protective mechanism and promote a malignant transformation. The relationship between melanocytes and keratinocytes—specifically, those mediated by paracrine factors—is not completely known. Our data suggested that keratinocyte-derived factors prevented or facilitated the escape of OIS in melanocytes and contributed to continued proliferation of the transformed melanocytes. Understanding what factors might be preventing or reversing senescence might help to identify therapeutic targets to prevent melanomagenesis. Recent studies have shown the effects of c-Myc-activated *USP-AS1* [43] and *ZNF* [43] in bypassing senescence and promoting tumorigenesis. Thus, it would be interesting to evaluate whether keratinocytes enable the secretion of these factors in the presence of oncogenic melanocytes and/or by themselves. Finally, we documented the differences in the secretome of Caucasian and African-American melanocytes both in isolation and in the presence of autologous and allogeneic keratinocytes. Our results showed the effect of a *BRAF* mutation on VEGF-A secretion, as previously shown in other tumors such as papillary thyroid cancer [44]. This observation suggested that upon an oncogenic insult (i.e., a *BRAF* mutation), Caucasian melanocytes were no longer dependent on the primary microenvironment for angiogenesis. Our data sheds light on the potential influence of keratinocytes in the disparity in the incidences of melanoma in Caucasian and African-American populations. It has been previously shown that VEGF-A is secreted in higher amounts by Vemurafenib (a *BRAF^V600E^* inhibitor)-resistant cell lines compared with its Vemurafenib-sensitive counterparts [40]. This leads us to posit that the pharmacological inhibition of mutant *BRAF* is unlikely to have an effect on VEGF-A secretion, at least in the latter stages of melanomagenesis. Further studies are needed to establish a causal link between the pharmacological inhibition of *BRAF^V600E^* and an increased vascularization. Similarly, further experiments are needed to investigate the effect of the pharmacological inhibition of the MAP kinase pathway and LIF secretion. In the context of angiogenesis, the role of matrix metalloproteinases may also be of interest. Although most MMPs are known to primarily play a role in invasion and metastasis, several MMPs may have anti-tumor properties. For example, a higher *MMP-8* was found in the serum of melanoma patients with highly vascularized primary tumors, suggesting a pro-tumorigenic function [45]. Similarly, the expression of *MMP-1* was reported to promote anchorage-independent growth and melanoma growth through the generation of active transforming growth factor beta [46]. 

## 4. Materials and Methods

### 4.1. Microdevice Fabrication and Operation

We fabricated the microfluidic devices by soft lithography as described in [17]. Briefly, we spun multiple SU-8 layers with the thickness desired and sequentially exposed them to UV light through a photomask. This process generated an SU-8 wafer with the desired geometry. Liquid PDMS was mixed with the curing agent at a 1:10 ratio; this mixture was poured and polymerized on the SU-8 wafer. The polymerized PDMS was then peeled off and plasma-bonded to a petri dish. One of our microdevice designs included a central circular microchamber to culture melanocytes with two lateral chambers on the side to seed dermal fibroblasts and/or keratinocytes. The central chamber was connected to the lateral chambers by a series of concentric narrow microchannels (i.e., 10 μm height). The microdevice was designed to confine the different cell populations in their respective chambers (i.e., lateral vs. central), allowing cellular crosstalk and migration. The second device that we used consisted of five parallel chambers delimited by a series of trapezoid-shaped pillars. The chambers adjacent to the extreme right and left chambers were filled with collagen and had pillars on either side to prevent the transfer of cells across the chambers. The central chamber was seeded with melanocytes and the extreme lateral chambers were seeded with keratinocytes.

### 4.2. Cell Culture

Primary melanocytes, keratinocytes and dermal fibroblasts were isolated from fresh neonatal foreskin specimens obtained from a local birthing unit (Meriter Hospital, Madison, WI, USA). Melanocytes were cultured in a Gibco 254 medium supplemented with a human melanocyte growth supplement (ThermoFisher, Waltham, MA, USA). Keratinocytes were cultured in a Gibco 154 medium supplemented with a human keratinocyte growth serum (ThermoFisher, Waltham, MA, USA) and 1% pen/strep. All cells in the microdevice were cultured in a 1:1 ratio of melanocyte and keratinocyte media. For the European or African genetic ancestry analysis, DNA samples from human neonatal skin fibroblasts were isolated and genotyped using a panel of 39 ancestry informative marker single nucleotide polymorphism assays (AIM-SNP) at the Centre for Human Genetics of Marshfield Clinic, WI. The Stephens and Pritchard code (2003) (version 2.3.4) was used to determine the genetic ancestry and admixture estimates [47]. To prevent melanocytes and keratinocytes from mixing in the microdevice, we used 2.5 mg/mL collagen (Collagen Type I, Corning Inc. Catalogue no. 354249, Corning, NY, USA) to confine the cells in their respective chambers.

### 4.3. Lentivirus Preparation and Transduction

Lentiviruses were prepared as described previously [48]. Briefly, HEK293 cells were plated on 10 cm plates in DMEM and 10% FBS without antibiotics and allowed to reach a 90% confluence within 24–48 h. Following the use of a Lipofectamine 2000 (Thermo Fisher, Waltham, MA, USA, #11668019), the protocol cells were then triple co-transfected with plasmids containing the packaging (psPAX2, Addgene #12260) and a VSV G envelope (pMD2.G, Addgene #12259, Watertown, MA, USA) as well as the target genes *BRAF^V600E^-GFP* (a gift from Dr. Raabe [49]) or empty vector-GFP (a gift from Dr. Herlyn [50]). A quantity of the lentiviruses was titered using the QuickTiter Lentivirus Titer Kit (Cell Biolabs Inc. Catalogue no. VPK-107, San Diego, CA, USA) according to the manufacturer’s instructions. Virus preparations with >10^8^ titer units/mL (TU/mL) were selected for the transduction. For the transduction, melanocytes were seeded on 6 cm plates and lentiviruses were added along with polybrene (5 µg/mL, Santacruz, Dallas, TX, USA) to enhance the transduction efficiency. Multiple doses of the lentiviruses were added over 2 days to ensure a maximal transduction efficiency.

### 4.4. Senescence-Associated Beta-Galactosidase Analysis

For the fluorescence analysis of the senescence-associated beta-gal (SA-β-gal), BioTrackerTM DCM-β-gal Live Cell Dye (Sigma Aldrich, St. Louis, MO, USA, Cat. # SCT050) was used according to the manufacturer’s instructions. For the colorimetric analysis of SA-β-gal, a senescence β-galactosidase staining kit (Cell Signaling Technology, Davers, MA, USA, Cat. # 9860) was used according to the manufacturer’s instructions.

### 4.5. Immunofluorescence

The cells were plated on 24-well plates, fixed in 4% formalin for 15 min and then incubated in a permeabilization/blocking buffer consisting of 0.3% Triton X-100, 1% BSA and 4% normal horse serum in PBS for 60 min (0.5 mL/well of a 24-well plate). Primary antibodies (p16: Cell Signalling Technology, Danvers, MA, USA, Catalogue No. D7C1M; Ki67: AbCam, Cambridge, UK, Catalogue No. ab16667) were diluted in a blocking buffer (1:2000) and incubated overnight at 4 °C. The following day, the cells were washed 3 times with PBST (1% Tween-20 in PBS) for 5 min and incubated for 1 h with a secondary antibody (Vector Biolabs, Malvern, PA, USA, anti-mouse IgG, Catalogue No. DI-1794) at room temperature. After three washes with PBST, Hoechst was added to the cells and incubated for 15 min for nuclear staining. The cells were washed thrice with BSA/PBS and visualized using an EVOS microscope (Thermo Fisher, Waltham, MA, USA). 

### 4.6. RNA Isolation and qPCR

Melanocytes in the central chamber of the device were detached using 0.05% trypsin. RNA was extracted using a Dynabeads™ mRNA purification kit (Thermo Fisher, Waltham, MA, USA, catalogue no. 61006) according to the manufacturer’s instructions. The RNA was converted to cDNA using a RT2 first strand kit (Qiagen, Germantown, MD, USA, Cat. No./ID: 330401) and amplified using a Qiagen RT2 PreAMP cDNA synthesis kit (Qiagen, Cat. No./ID: 330451). For the pre-amplification step, Qiagen RT^2^ PreAMP cDNA synthesis primer mix for a human cellular senescence PCR array (330241) was used. The Qiagen human cellular senescence RT2 profiler array (Qiagen ID: PAHS-050Z) was used to evaluate the changes in the gene expression.

### 4.7. Multiplexed Assay for the Measurement of Growth Factor Secretion

Keratinocytes were cultured for 3 days to generate the keratinocyte-conditioned medium. Transformed melanocytes were seeded on a 24-well plate after the keratinocyte-conditioned medium was added. Melanocytes were cultured in the conditioned medium for 3 days, after which they were harvested to analyze the growth factor secretion. To measure the growth factors secreted by melanocytes in the presence of the keratinocyte-conditioned medium, a multiplexed bead-based assay was used. Growth factors were analyzed using a Growth Factor 11-Plex Human ProcartaPlex™ panel (Thermo Fisher, Waltham, MA, USA, EPX110-12170-901) following the manufacturer’s guidelines. Briefly, the growth factors were detected by sequentially incubating them with the antibody bead cocktail solution, the detection antibody and streptavidin–R-phycoerythrin. The magnetic beads were washed using a magnetic plate washer prior to mixing with the samples and after each incubation. The samples were read on a MAGPIX Luminex Xmap system (Luminex Corporation, Austin, TX, USA) using Luminex xPonent software. The results were expressed as the mean fluorescence intensity (MFI) for each analyte in each sample. The average MFI values from the standards were converted to concentrations (pg/mL) using cytokine-specific standard curve data.

### 4.8. Images and Statistical Analysis

Images were processed in FIJI and the parameters were kept constant between the control and sample images. The statistical analyses were performed using GraphPad Prism 8. A Student’s unpaired *t*-test and a one-way ANOVA test were performed for significance studies with a 95% confidence interval (*p* < 0.05). All cells used in this study were primary cells obtained from donors according to the guidelines of the Declaration of Helsinki and approved by the Institutional Review Board of UnityPoint Health–Meriter (a partner of UW Health) (Protocol #2006-006; original approval 5 April 2007 and current approval 22 December 2021). This protocol falls under the waiver of consent due to the limited PHI collected and the anonymity of the subject. All the experiments were repeated at least 3 independent times and we combined multiple African-American and Caucasian donors to account for a donor-to-donor variability.

## 5. Conclusions

Further studies are needed to test if forced changes in the expression of genes and factors we identified in oncogenic melanocytes could reverse the senescence phenotype in the absence of keratinocytes. Additional factors regulated by the microenvironment such as *MDM4* and β-catenin could be analyzed by an ELISA and their roles in affecting oncogene-induced senescence in the transformed melanocytes could be investigated. Such studies could help devise novel strategies for melanoma prevention and reverse the increasing trend of melanoma incidence.

## Data Availability

The data presented in this study are available in the manuscript file and the Supplementary Information file. If additional data are required, contact the corresponding authors.

## Supplementary Materials

The following supporting information can be downloaded at: https://www.mdpi.com/article/10.3390/cancers14051233/s1. Figure S1: (A) BRAFV600E transformation induces p27 expression in melanocytes. Left image shows control normal melanocytes and right image shows BRAFV600E transformed melanocytes with increased p27 expression as shown by immunofluorescence. (B) Senescence Associated Heterochromatin Foci. BRAFV600E melanocytes show punctate staining in nuclei due to the formation of the SAHF. Control melanocytes show a diffused staining of the nuclei, Figure S2: (A) Co-culture of Keratinocytes with BRAFV600E melanocytes. Keratinocytes were co-cultured with empty vector control (Empty Vector) and transformed melanocytes (BRAFV600E) seeded on a 24 well plate in a cell culture insert, for 1 week. Figures show SABG analysis of cells with (Co-Culture) and without (Monoculture) keratinocytes. Presence of keratinocytes lowers the number of SABG positive cells. Quantification of senescence on the right image. (B) Co-Culture of Keratinocytes with BRAFV600E melanocytes. Keratinocytes were co-cultured with transformed melanocytes (BRAFV600E) seeded on a 24 well plate in a cell culture insert, for 1 week. Figures p16 immunostaining of cells with (Co-Culture) and without (Monoculture) keratinocytes. Presence of keratinocytes lowers the number of p16 positive cells. Quantification of p16 expression on the right image. (C) Co-Culture of Fibroblasts with BRAFV600E melanocytes. A. Fibroblasts were co-cultured with transformed melanocytes seeded on a 24 well plate in a cell culture insert, for 1 week. Figures show SABG analysis of cells with (+) and without (−) fibroblasts. Presence of fibroblasts lowers the number of SABG positive cells. Quantification of morphology change as percentage of round cells shown on right. *, **, ***, and **** denote *p*-value < 0.05, 0.01, 0.005, and 0.001 respectively, Figure S3: Fold Change of genes CDKN2C, TGFB1, and TBX2 in the presence (+) and absence (−) of keratinocytes. *, **, and ***, denote *p*-value < 0.05, 0.01, and 0.005 respectively, Figure S4: Secretion of VEGF-A and LIF by Kerationcytes. Graph showing amount of VEGF-A and LIF secreted by Caucasian and African American keratinocytes in different intervals. NK103 indicates Caucasian keratinocytes and NK 20 indicates African American keratinocytes. (0–3) represents VEGF-A/LIF in the media built up since seeding of keratinocytes until day 3 without any change of media in between. (3–6) represents VEGF-A/LIF in the media after from day 3 (post media change) to day 6. (0–6) represents VEGF-A/LIF in the media built up since seeding of keratinocytes until 6 days without any change of media in between. *, **, and ***, denote *p*-value < 0.05, 0.01, and 0.005 respectively.
