# Supplementary material for: Role of the Skin Microenvironment in Melanomagenesis: Epidermal Keratinocytes and Dermal Fibroblasts Promote BRAF Oncogene-Induced Senescence Escape in Melanocytes
Source: Cancers (Basel). 2022 Feb 27;14(5):1233. doi: 10.3390/cancers14051233 (PMC8909265; doi:10.3390/cancers14051233)
Supplement: Supplementary file 1 [file cancers-14-01233-s001.zip › cancers-1555988-supplementary.pdf]

cancers

MDPI

Supplementary Materials

Role of the Skin Microenvironment in Melanomagenesis: Epidermal Keratinocytes and Dermal Fibroblasts Promote BRAF Oncogene-Induced Senescence Escape in Melanocytes

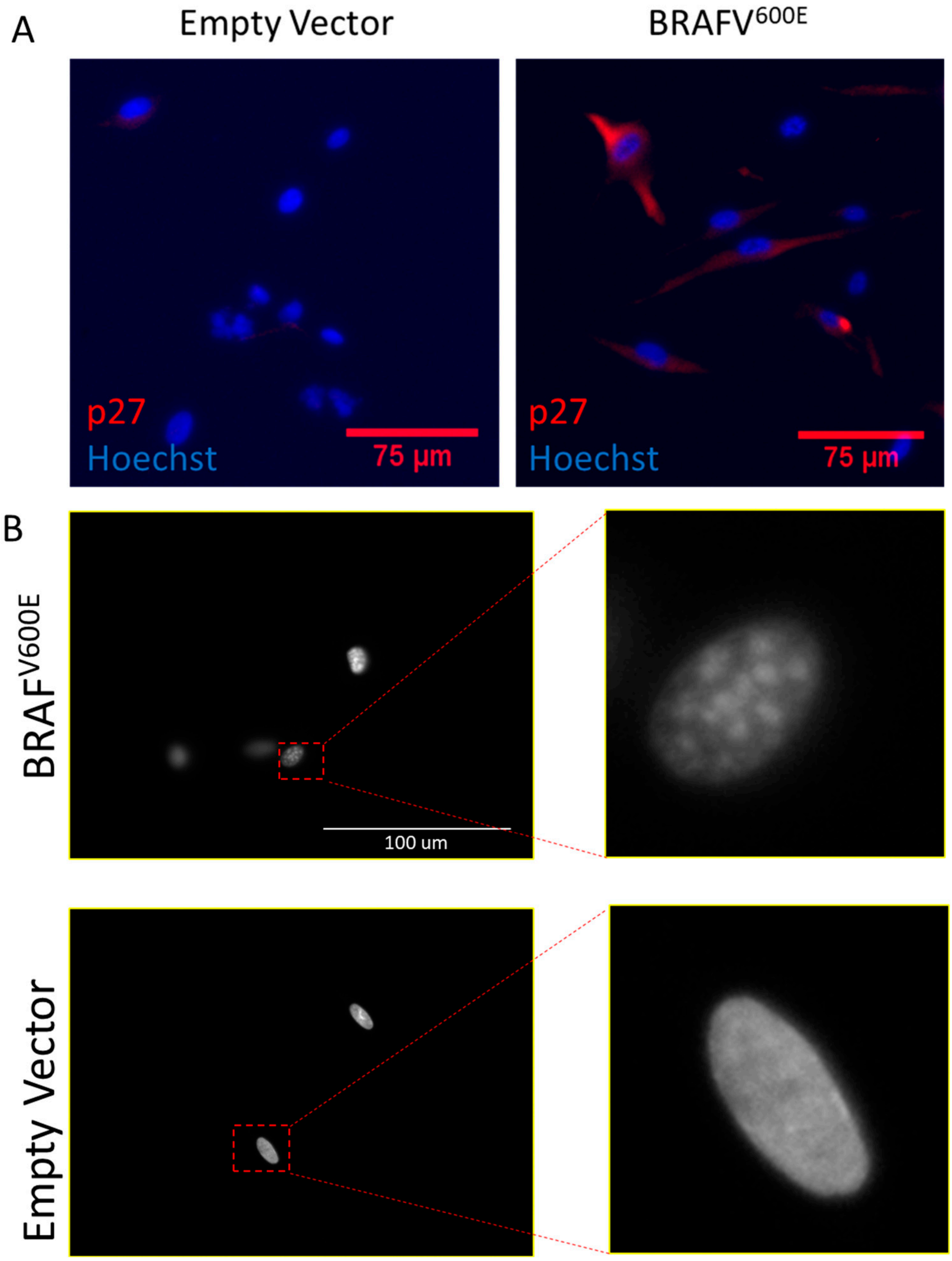

Figure S1. (**A**) BRAF^{V600E} transformation induces p27 expression in melanocytes. Left image shows control normal melanocytes and right image shows BRAF^{V600E} transformed melanocytes with increased p27 expression as shown by immunofluorescence. (**B**) Senescence Associated Heterochromatin Foci. BRAF^{V600E} melanocytes show punctate staining in nuclei due to the formation of the SAHF. Control melanocytes show a diffused staining of the nuclei.

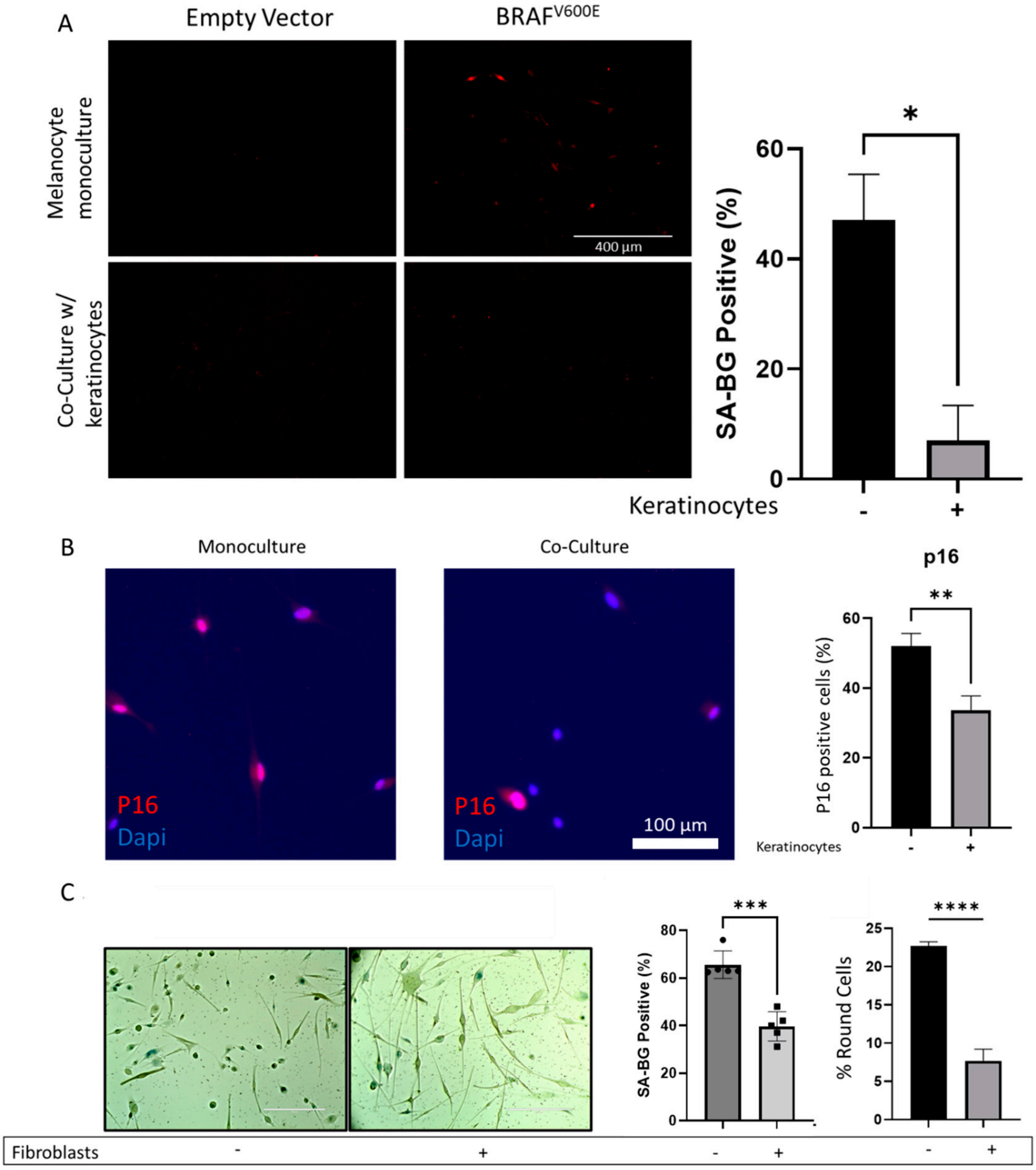

Figure S2. (**A**) Co-culture of Keratinocytes with BRAFV600E melanocytes. Keratinocytes were co-cultured with empty vector control (Empty Vector) and transformed melanocytes ($BRAF^{V600E}$) seeded on a 24 well plate in a cell culture insert, for 1 week. Figures show SABG analysis of cells with (Co-Culture) and without (Monoculture) keratinocytes. Presence of keratinocytes lowers the number of SABG positive cells. Quantification of senescence on the right image. (**B**) Co-Culture of Keratinocytes with BRAFV600E melanocytes. Keratinocytes were co-cultured with transformed melanocytes (BRAFV600E) seeded on a 24 well plate in a cell culture insert, for 1 week. Figures p16 immunostaining of cells with (Co-Culture) and without (Monoculture) keratinocytes. Presence of keratinocytes lowers the number of p16 positive cells. Quantification of p16 expression on the right image. (**C**) Co-Culture of Fibroblasts with BRAFV600E melanocytes. A. Fibroblasts were co-cultured with transformed melanocytes seeded on a 24 well plate in a cell culture insert, for 1 week. Figures show SABG analysis of cells with (+) and without (−) fibroblasts. Presence of fibroblasts lowers the number of SABG positive cells. Quantification of morphology change as percentage of round cells shown on right. *, **, ***, and **** denote *p*-value < 0.05, 0.01, 0.005, and 0.001 respectively

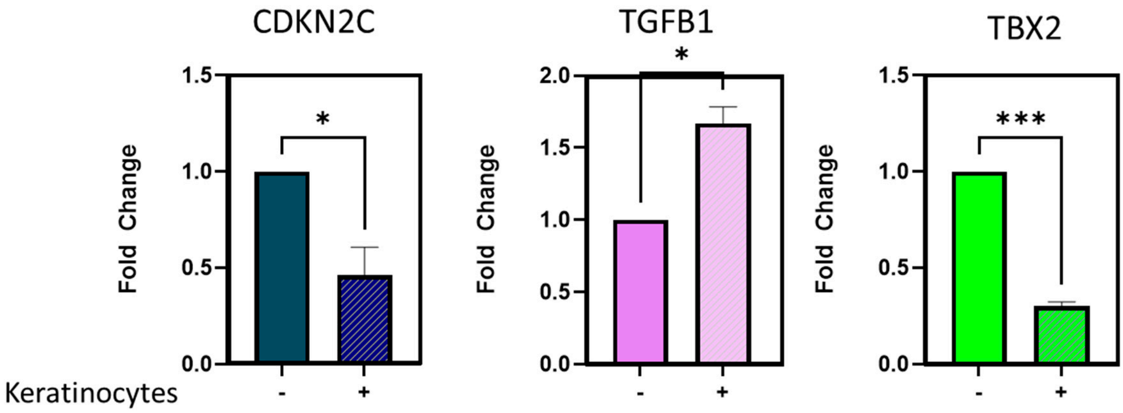

Figure S3. Fold Change of genes CDKN2C, TGFB1, and TBX2 in the presence (+) and absence (−) of keratinocytes. *, and ***, denote *p*-value < 0.05, and 0.005 respectively.

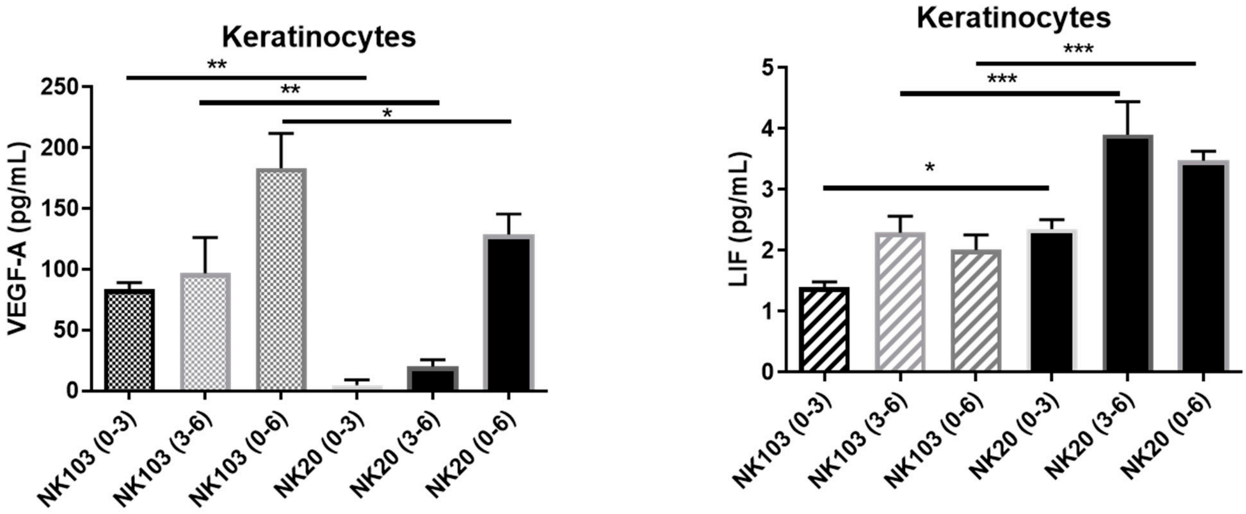

Figure S4. Secretion of VEGF-A and LIF by Kerationcytes. Graph showing amount of VEGF-A and LIF secreted by Caucasian and African American keratinocytes in different intervals. NK103 indicates Caucasian keratinocytes and NK 20 indicates African American keratinocytes. (0-3) represents VEGF-A/LIF in the media built up since seeding of keratinocytes until day 3 without any change of media in between. (3–6) represents VEGF-A/LIF in the media after from day 3 (post media change) to day 6. (0–6) represents VEGF-A/LIF in the media built up since seeding of keratinocytes until 6 days without any change of media in between. *, **, and ***, denote *p*-value < 0.05, 0.01, and 0.005 respectively.
